# Normal *ATXN3* Allele but Not *CHIP* Polymorphisms Modulates Age at Onset in Machado–Joseph Disease

**DOI:** 10.3389/fneur.2012.00164

**Published:** 2012-11-19

**Authors:** Marcondes C. França, Vanessa E. Emmel, Anelyssa D’Abreu, Cláudia V. Maurer-Morelli, Rodrigo Secolin, Luciana Cardoso Bonadia, Marilza Santos da Silva, Anamarli Nucci, Laura Bannach Jardim, Maria Luiza Saraiva-Pereira, Wilson Marques, Henry Paulson, Iscia Lopes-Cendes

**Affiliations:** ^1^Department of Neurology, University of CampinasCampinas, Brazil; ^2^Laboratory of Genetic Identification, Hospital de Clínicas de Porto AlegrePorto Alegre, Brazil; ^3^Department of Medical Genetics, University of CampinasCampinas, Brazil; ^4^Department of Internal Medicine, Universidade Federal do Rio Grande do SulPorto Alegre, Brazil; ^5^Instituto Nacional de Ciência e Tecnologia de Genética Médica PopulacionalPorto Alegre, Brazil; ^6^Department of Biochemistry, Universidade Federal do Rio Grande do SulPorto Alegre, Brazil; ^7^Department of Neurology, University of Sao Paulo at Ribeirão PretoRibeirão Preto, Brazil; ^8^Department of Neurology, University of MichiganAnn Arbor, MI, USA

**Keywords:** SCA3, Machado–Joseph disease, polyQ, modifier genes, age at onset

## Abstract

**Background:** Age at onset (AO) in Machado–Joseph disease (MJD) is closely associated with the length of the CAG repeat at the mutant *ATXN3* allele, but there are other intervening factors. Experimental evidence indicates that the normal *ATXN3* allele and the C-terminal heat shock protein 70 (Hsp70)-interacting protein (*CHIP*) may be genetic modifiers of AO in MJD. **Methods:** To investigate this hypothesis, we determined the length of normal and expanded CAG repeats at the *ATXN3* gene in 210 unrelated patients with MJD. In addition, we genotyped five single nucleotide polymorphisms (SNPs) within the *CHIP* gene. We first compared the frequencies of the different genotypes in two subgroups of patients who were highly discordant for AO after correction for the length of the expanded CAG allele. The possible modifier effect of each gene was then evaluated in a stepwise multiple linear regression model. **Results:** AO was associated with the length of the expanded CAG allele (*r*^2^ = 0.596, *p* < 0.001). Frequencies of the normal CAG repeats at the *ATXN3* gene and of *CHIP* polymorphisms did not differ significantly between groups with highly discordant ages at onset. However, addition of the normal allele improved the model fit for prediction of AO (*r*^2^ = 0.604, *p* = 0.014). Indeed, we found that the normal CAG allele at *ATXN3* had a positive independent effect on AO. **Conclusion:** The normal CAG repeat at the *ATXN3* gene has a small but significant influence on AO of MJD.

## Introduction

Machado–Joseph disease [MJD, also named spinocerebellar ataxia (SCA) type 3 (SCA3)], is the most common autosomal dominant SCA worldwide (Schols et al., 2004). It is caused by an unstable trinucleotide (CAG) repeat expansion in exon 10 of the *ATXN3* gene, which leads to an elongated polyglutamine (PolyQ) tract in the encoded protein, ataxin-3 (ATXN3; Kawaguchi et al., 1994). MJD belongs to a group of neurodegenerative disorders (polyQ diseases) caused by these unstable mutations, such as SCAs 1, 2, 6, 7, and Huntington disease (HD; Everett and Wood, 2004). These disorders usually begin in early adulthood and slowly progress over time, and there is significant phenotypic variability (Everett and Wood, 2004; Schols et al., 2004).

The length of the expanded allele in polyQ disorders is closely associated with age at onset (AO) of symptoms. In MJD, previous data indicate that the ATXN3 expansion explains 50–80% of AO variance (Maciel et al., 1995; Dürr et al., 1996; van de Warrenburg et al., 2005). Additional environmental and/or genetic factors must exist that account for the remaining variability. In other polyQ disorders, some modifier loci and genes have already been identified (Rubinsztein et al., 1997; Hayes et al., 2000; Pulst et al., 2005; Metzger et al., 2008). This motivated us to investigate two possible genetic modifiers of AO in a large sample of unrelated Brazilian patients with MJD, C-terminal heat shock protein 70 (Hsp70)-interacting protein (*CHIP*), and the normal *ATXN3* allele.

C-terminal heat shock protein 70 (Hsp70)-interacting protein is a ubiquitin ligase that was recently shown to suppress polyQ aggregation in polyQ disorders (Miller et al., 2005; Al-Ramahi et al., 2006). In cellular and animal MJD models, *CHIP* under expression aggravates disease phenotype (Miller et al., 2005; Williams et al., 2009). Regarding the potential influence of normal ATXN3, there are conflicting results about its role as an independent modifier of AO in MJD (Dürr et al., 1996; van de Warrenburg et al., 2002). Recent evidence, however, suggests that it interacts with its mutant counterpart, and this might be relevant in the pathogenic cascade of neurodegeneration (Jia et al., 2008).

## Materials and Methods

### Subject selection

Two-hundred and ten patients from unrelated families and with molecular confirmation of MJD were included in the present study. They were recruited from three neurogenetic centers in southern Brazil: University of Campinas (UNICAMP), University of Sao Paulo at Ribeirao Preto (USP-RP), and Federal University of Rio Grande do Sul (UFRGS). This study was approved by our institution Ethics Committee and written informed consent was obtained from all participants.

Age at onset was defined as the age at which the first symptoms of ataxia began. This was estimated according to the reports of patients and close relatives. Individuals with autosomal dominant SCA but no molecular confirmation of MJD and those without available data about AO were excluded from the study.

### Molecular studies

#### Normal and expanded ATXN3 alleles

Genomic DNA (gDNA) was extracted from lymphocytes in peripheral blood following standard techniques (Sambrook et al., 1989). We performed polymerase chain reaction (PCR) to determine the length of the normal and expanded alleles at exon 10 of *ATXN3*. The final volume for each assay was 10 μl: 50 ng of gDNA; 125 μM of each dNTP (dATP, dCTP, dGTP, dTTP); 2.5 pmol of each primer; Tris-HCl 20 mM; MgCl_2_ 1.75 mM; KCl 50 mM; and 1.5 Units of Taq DNA polymerase. PCR products were resolved using capillary electrophoresis on MegaBACE™ 1000 96-capillary sequencers (GE Healthcare, Buckinghamshire, UK) or ABI3130*xl* (Applied Biosystems, Foster City, CA, USA) and length of (CAG) repeats determined by comparison with standard makers.

#### CHIP polymorphisms

We selected five single nucleotide polymorphisms (SNPs) in the *CHIP* gene, rs12599315, rs11558085, rs11861355, rs6597, and rs3204090, to genotype in our cohort of patients with MJD. These polymorphisms were chosen through SNP tagging by HAPLOVIEW software (Barrett et al., 2005), based on the four populations evaluated in the HapMap Project (The International HapMap Consortium, 2005). We used HAPLOVIEW to estimate minor allele frequency (MAF) and Hardy–Weinberg Equilibrium (HWE) for these SNPs. We used Real-Time PCR (Applied Biosystems, Foster City, CA, USA) to genotype these SNPs, and each reaction was performed in a final volume of 7:1 μl of DNA; 3.5 μl of TaqMan; 0.175 μl of SNP genotyping assay; and 2.325 μl of milliQ water.

### Statistical analysis

We used the G*Power v.3.1 (Faul et al., 2007) software to evaluate the *post hoc* statistical power of the sample to detect association with the following parameters: two-tail; effect size = 0.15; significance level α = 0.01 (corrected for multiple comparisons, since we analyzed five SNPs); total sample size = 210; number of predictors = 3.

We then examined the association between AO and the length of the expanded (CAG) using a linear regression model. This allowed us to determine the residual AO corrected for expanded (CAG) for each patient. We selected two groups of patients from the curve of residual AO, those with extremely early and late onset. These were defined as patients who had residual AO at least one standard deviation earlier and later than the mean AO, respectively. We compared the distribution of genotypes and alleles for the normal *ATXN3* allele and for *CHIP* polymorphisms in the two groups using the Mann–Whitney *U* test. Subsequently, we examined whether the addition of the genotypes for each candidate gene as independent variables improved the AO vs. the expanded (CAG) model in a stepwise regression analysis. Statistical analysis was performed on SYSTAT 10.2. *p* Values < 0.05 were considered significant.

## Results

**Figure 1:**
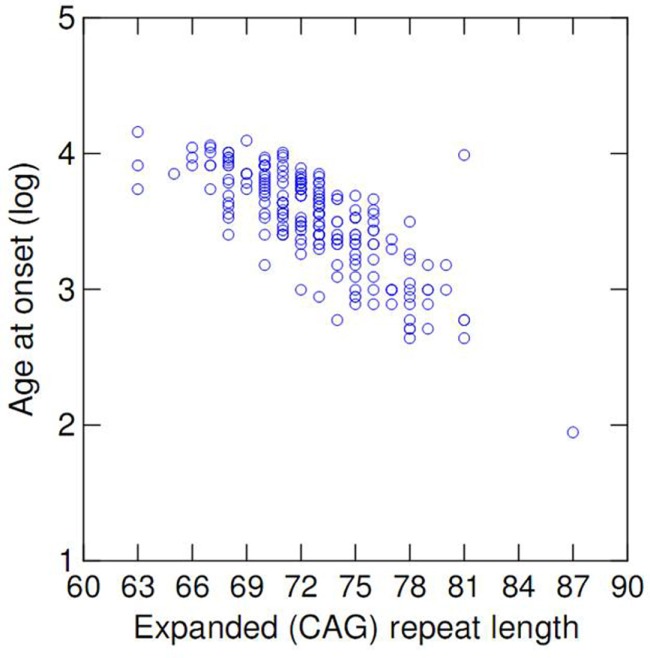
**Scatterplot of age at onset vs. length of expanded (CAG) repeat at *ATXN3***.

The mean age of onset in the cohort was 35.7 (range 7–64, SD = 11.3) years. The mean length of expanded and normal (CAG) repeats was 72 (ranging from 63 to 87, SD = 3.6) and 21 (range 13–37, SD = 5.3), respectively. There was a significant negative correlation of AO and the (CAG) repeat expansion (Pearson *r*^2^ = 0.573, *p* < 0.001 – Table 1). Logarithmic transformation of AO led to a slightly better fit [log(*r*^2^) = 0.596, *p* < 0.001], and thus log-transformed AO was used in all further comparative analyses (Figure 1).

**Table 1:** **Predicted ages at onset for each expanded (CAG) length at the mutant allele in MJD**.

Expanded CAG	AO	95% CI
60	88	79–98
61	81	73–90
62	76	69–83
63	70	64–76
64	65	60–70
65	60	56–65
66	56	52–59
67	52	49–55
68	48	46–50
69	44	42–46
70	41	40–43
71	38	37–39
72	35	34–36
73	33	32–34
74	30	29–31
75	28	27–29
76	26	25–27
77	24	23–25
78	22	21–24
79	21	19–22
80	19	18–21
81	18	16–19
82	17	15–18
83	15	14–17
84	14	13–16
85	13	12–15
86	12	11–14
87	11	10–13
88	10	9–12
89	10	8–11
90	9	8–10

The SNP average genotype call rate was 100% and the average genotyping success rate of sample subjects was 100%. Three *CHIP* SNPs presented MAF < 0.05, and one was not under HWE (*p* < 0.001); therefore these four SNPs were excluded from further analysis (Table 2).

**Table 2:** **SNPs from CHIP gene evaluated in this cohort**.

SNP	Position Ch16 (bases)	MAF	HWE *p*-value
rs12599315	729831	0.281	<0.001*
rs11558085	730620	<0.05*	<0.001*
rs11861355	731517	<0.05*	<0.001*
rs6597	731725	0.136	0.345
rs3204090	732465	<0.05*	<0.001*

*These SNPs were removed from further analyses due to MAF and/or HWE status.

**Figure 2:**
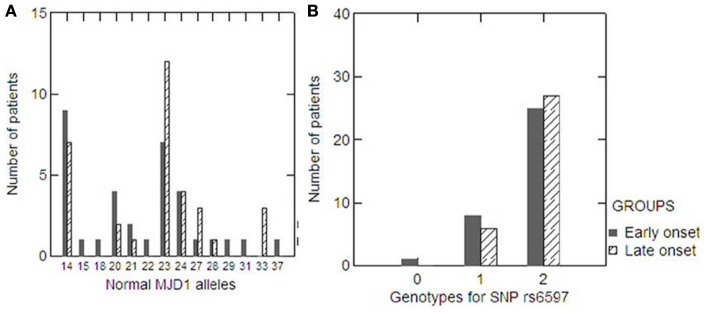
**Distribution of normal *ATXN3* (CAG) repeat alleles in the extremely early (black column) and late (dashed column) onset groups (A)**. Distribution of genotypes for the SNP rs6597 within the *CHIP* gene in the extremely early (black column) and late (dashed column) onset groups **(B)**.

Statistical power analysis revealed that the power of our sample to detect a genetic association was 99.86%. According to residual AO corrected for expanded (CAG) repeats, there were 34 and 33 patients in the extremely early and late onset groups, respectively. The mean AO differed significantly between groups (26.4 ± 8.4 vs. 48.7 ± 6.3, Mann–Whitney *p* < 0.001), but there was no significant difference in the length of the (CAG) repeat expansion (72 ± 3.7 vs. 71 ± 3.0, Mann–Whitney *p* = 0.350). The distribution of normal *ATXN3* (CAG) repeat alleles was also similar in the two groups (Mann–Whitney *p* = 0.217, Figure 2A). Allele and genotype frequencies for rs6597 did not differ between the two groups (Mann–Whitney *p* = 0.392 and 0.435, respectively; Figure 2B).

The inclusion of the normal *ATXN3* allele as an independent variable in the log-transformed AO vs. expanded (CAG) model resulted in a small but significant improvement in the association (*r*^2^ = 0.601, *p* = 0.034; Table 3). In this combined model, patients with identical lengths of the (CAG) expansion at the mutant allele but different lengths at the normal allele would have predicted AO separated by up to 7 years (Table 4). In contrast, the addition of rs6597 genotypes as independent variables in the previous model did not yield a better fit – Table 3.

**Table 3:** **Multiple linear regression analyses of possible candidate genetic modifiers of log-transformed age at onset in Machado–Joseph disease**.

Model	*r*^2^	Δ*r*^2^	*p*-Value
MJD (CAG) exp	0.596	−	<0.001
MJD (CAG) exp + normal (CAG)	0.601	0.05	0.034
MJD (CAG) exp + CHIP rs6597 genotype	0.594	0.0	0.452

**Table 4:** **Predicted ages at onset for each normal (CAG_10–4__0_) and expanded (CAG_60–9__0_) repeat length in MJD (MLE – maximum likelihood estimation method)**.

Expanded CAG	Normal CAG						
	10	15	20	25	30	35	40
60	85 (75–97)	86 (77–97)	88 (78–98)	89 (79–100)	90 (80–102)	92 (80–105)	93 (80–109)
61	79 (70–89)	80 (72–89)	81 (73–90)	82 (74–92)	84 (75–94)	85 (74–97)	86 (74–100)
62	73 (65–82)	74 (67–82)	75 (68–83)	76 (69–84)	77 (70–86)	79 (69–89)	80 (69–92)
63	68 (60–76)	69 (62–76)	70 (64–76)	71 (65–77)	72 (65–79)	73 (65–82)	74 (64–85)
64	63 (56–70)	64 (58–70)	65 (60–70)	66 (60–71)	67 (61–73)	68 (60–76)	69 (60–78)
65	58 (53–64)	59 (54–64)	60 (56–64)	61 (56–65)	62 (57–67)	63 (56–70)	64 (56–72)
66	54 (49–59)	55 (51–59)	55 (52–59)	56 (53–60)	57 (53–62)	58 (52–64)	59 (52–67)
67	50 (46–55)	51 (47–54)	51 (48–54)	52 (49–55)	53 (49–57)	54 (49–59)	55 (48–62)
68	46 (42–50)	47 (44–50)	48 (45–50)	48 (46–51)	49 (46–53)	50 (45–55)	51 (45–57)
69	43 (39–46)	43 (41–46)	44 (42–46)	45 (43–47)	45 (43–48)	46 (42–50)	47 (42–53)
70	40 (37–43)	40 (38–43)	41 (39–43)	41 (40–43)	42 (40–45)	43 (39–47)	43 (39–49)
71	37 (34–40)	37 (35–39)	38 (37–39)	38 (37–40)	39 (37–41)	40 (36–43)	40 (36–45)
72	34 (32–37)	35 (33–36)	35 (34–36)	36 (34–37)	36 (34–38)	37 (34–40)	37 (33–42)
73	32 (29–34)	32 (30–34)	33 (31–34)	33 (32–34)	34 (32–35)	34 (31–37)	35 (31–39)
74	29 (27–32)	30 (28–31)	30 (29–31)	31 (29–32)	31 (29–33)	32 (29–34)	32 (29–36)
75	27 (25–29)	28 (26–29)	28 (27–29)	28 (27–30)	29 (27–31)	29 (27–32)	30 (27–33)
76	25 (23–27)	25 (24–27)	26 (25–27)	26 (25–27)	27 (25–28)	27 (25–30)	27 (25–31)
77	23 (21–25)	24 (22–25)	24 (23–25)	24 (23–26)	25 (23–26)	25 (23–27)	25 (23–29)
78	22 (20–24)	22 (20–23)	22 (21–24)	23 (21–24)	23 (21–25)	23 (21–26)	24 (21–27)
79	20 (18–22)	20 (19–22)	21 (19–22)	21 (20–22)	21 (20–23)	22 (20–24)	22 (19–25)
80	19 (17–20)	19 (17–20)	19 (18–20)	19 (18–21)	20 (18–21)	20 (18–22)	20 (18–23)
81	17 (15–19)	17 (16–19)	18 (16–19)	18 (17–19)	18 (17–20)	18 (17–21)	19 (17–21)
82	16 (14–18)	16 (15–18)	16 (15–18)	17 (15–18)	17 (15–19)	17 (15–19)	17 (15–20)
83	15 (13–17)	15 (13–17)	15 (14–17)	15 (14–17)	16 (14–17)	16 (14–18)	16 (14–19)
84	14 (12–15)	14 (12–15)	14 (13–16)	14 (13–16)	14 (13–16)	15 (13–17)	15 (13–17)
85	13 (11–14)	13 (11–14)	13 (12–15)	13 (12–15)	13 (12–15)	14 (12–16)	14 (12–16)
86	12 (10–13)	12 (10–14)	12 (11–14)	12 (11–14)	12 (11–14)	13 (11–15)	13 (11–15)
87	11 (9–13)	11 (10–13)	11 (10–13)	11 (10–13)	12 (10–13)	12 (10–14)	12 (10–14)
88	10 (9–12)	10 (9–12)	10 (9–12)	11 (9–12)	11 (9–12)	11 (9–13)	11 (9–13)
89	9 (8–11)	9 (8–11)	10 (8–11)	10 (8–11)	10 (9–11)	10 (9–12)	10 (9–12)
90	9 (7–10)	9 (7–10)	9 (8–10)	9 (8–11)	9 (8–11)	9 (8–11)	9 (8–11)

## Discussion

Our results confirm the close association of AO and length of *ATXN3* (CAG) expansion in MJD, which is particularly evident among patients with large expansions. However, much of the AO variance cannot be explained by the (CAG) repeat expansion in the *ATXN3* gene, and thus other intervening factors, either environmental or genetic, must also contribute. In other polyQ disorders such as HD and SCA2, some genetic modifiers have already been identified, with individual contributions that account for 1–13% of unexplained AO variability (Rubinsztein et al., 1997; Hayes et al., 2000; Pulst et al., 2005; Metzger et al., 2008). Although DeStefano et al. (1996) reported that a familial factor independent of (CAG) repeat expansion influences AO in MJD, no such genetic modifier for MJD has been identified to date.

In the present study, there was no significant difference in allele and genotype frequencies for normal ATXN3 between patients with extremely late and early onset. This was possibly due to limitations in sample size in either group. However, regression analyses confirmed that the normal allele indeed has a small (around 1%) but significant modulating effect on AO. This can be fully appreciated by examining the data presented in Table 4, in which one can see that patients matched for (CAG) length at the mutant allele would have a clearly different predicted AO depending on the normal alleles they had inherited. Interestingly, this modulating effect of the normal ATXN3 allele is more relevant for patients with small expansions at the mutant allele (Table 4). Dürr et al. (1996) using a different approach, reported similar results in a cohort of French patients. There is also some evidence that the normal allele modifies AO in other polyQ disorders, such as SCA1 and SCA6 (van de Warrenburg et al., 2005). The normal *HTT* allele was also recently shown to interact with the expanded allele to determine AO in patients with HD (Aziz et al., 2009). In contrast, authors of a survey in the Netherlands did not replicate these findings (van de Warrenburg et al., 2002). That study, however, may have been underpowered to detect such small modifier effects.

The association of AO and the normal *ATXN3* (CAG) repeat allele suggests that this allele might play a modulatory role in disease pathogenesis. Although speculative, this possibility is supported by some experimental and pathologic data. Normal ATXN3 is found in the intraneuronal inclusions characteristic of MJD (Rüb et al., 2008). Furthermore, cell models in polyQ disorders have shown that normal proteins interact with the mutant counterparts in a polyQ length-dependent manner and that the rate of co-aggregate formation (normal + mutant) is proportional to the polyQ stretch in either the normal or expanded allele (Slepko et al., 2006). In this scenario, normal ATXN3 with longer polyQ chains could associate more efficiently with mutant ATXN3. Such an effect would sequestrate higher amounts of the mutant protein into the inclusion bodies and avoid its aberrant interactions with other proteins, thus slowing the neurodegenerative cascade. Indeed, this pathogenetic model would account for our finding that the association of (CAG) expansion at the normal allele and AO is positive.

C-terminal heat shock protein 70 (Hsp70)-interacting protein is a small protein that acts as a ubiquitin ligase and a co-chaperone in neurons (Miller et al., 2005; Al-Ramahi et al., 2006). It thus links two important mechanisms involved in protein quality control, the ubiquitin proteasome system and the molecular chaperones; these mechanisms have been increasingly implicated in the pathogenesis of polyQ diseases (Orr and Zoghbi, 2007). Evidence from cellular and animal models of MJD indicates that reduction or elimination of *CHIP* aggravates disease course and neuropathological abnormalities (Miller et al., 2005; Williams et al., 2009). In the face of these results, *CHIP* was the second candidate gene to be evaluated. We were left with a single *CHIP* SNP, which did not influence AO in our MJD cohort. Despite this, we believe that rs 6597 provides meaningful information with which to establish or refute the association because *CHIP* is a small gene encompassed by a unique haplotype block in the populations studied by the HapMap project. This result apparently contrasts with experimental data. A possible explanation is that *CHIP* plays an important role in the steps of MJD pathogenesis occurring after disease onset. An alternative explanation is that this cohort may just not have sufficient and functionally significant variation in *CHIP* to be detectable by genetic analysis.

We used two different statistical approaches to test whether the normal ATXN3 allele and AO are independently associated. Although the analysis of extremely early and late onset groups failed to demonstrate significance, the stepwise regression analyses showed an association. This result indeed indicates that the normal allele modifies AO, and this effect is independent of the expanded allele effect. In contrast, we were unable to show an association of *CHIP* polymorphisms with AO. Further research will be useful to determine whether these findings also hold in individuals with different ethnic backgrounds and to identify other modifier genes.

## Conflict of Interest Statement

The authors declare that the research was conducted in the absence of any commercial or financial relationships that could be construed as a potential conflict of interest.
